# Computer vision digitization of smartphone images of anesthesia paper health records from low-middle income countries

**DOI:** 10.1186/s12859-024-05785-8

**Published:** 2024-05-07

**Authors:** Ryan D. Folks, Bhiken I. Naik, Donald E. Brown, Marcel E. Durieux

**Affiliations:** 1 Department of Anesthesiology, University of Virginia, Charlottesville, VA USA; 2 School of Data Science, University of Virginia, Charlottesville, VA USA

**Keywords:** Computer vision, Computer extraction of time series, Document analysis

## Abstract

**Background:**

In low-middle income countries, healthcare providers primarily use paper health records for capturing data. Paper health records are utilized predominately due to the prohibitive cost of acquisition and maintenance of automated data capture devices and electronic medical records. Data recorded on paper health records is not easily accessible in a digital format to healthcare providers. The lack of real time accessible digital data limits healthcare providers, researchers, and quality improvement champions to leverage data to improve patient outcomes. In this project, we demonstrate the novel use of computer vision software to digitize handwritten intraoperative data elements from smartphone photographs of paper anesthesia charts from the University Teaching Hospital of Kigali. We specifically report our approach to digitize checkbox data, symbol-denoted systolic and diastolic blood pressure, and physiological data.

**Methods:**

We implemented approaches for removing perspective distortions from smartphone photographs, removing shadows, and improving image readability through morphological operations. YOLOv8 models were used to deconstruct the anesthesia paper chart into specific data sections. Handwritten blood pressure symbols and physiological data were identified, and values were assigned using deep neural networks. Our work builds upon the contributions of previous research by improving upon their methods, updating the deep learning models to newer architectures, as well as consolidating them into a single piece of software.

**Results:**

The model for extracting the sections of the anesthesia paper chart achieved an average box precision of 0.99, an average box recall of 0.99, and an mAP0.5-95 of 0.97. Our software digitizes checkbox data with greater than 99% accuracy and digitizes blood pressure data with a mean average error of 1.0 and 1.36 mmHg for systolic and diastolic blood pressure respectively. Overall accuracy for physiological data which includes oxygen saturation, inspired oxygen concentration and end tidal carbon dioxide concentration was 85.2%.

**Conclusions:**

We demonstrate that under normal photography conditions we can digitize checkbox, blood pressure and physiological data to within human accuracy when provided legible handwriting. Our contributions provide improved access to digital data to healthcare practitioners in low-middle income countries.

## Background

Globally, approximately 313 million surgical cases are performed annually. 6% of these surgeries are performed in low-middle income countries (LMICs), where a third of the global population currently resides. Surgical mortality rates are twice as high in LMICs, compared to high-income countries despite patients being younger, having a lower risk profile and undergoing less invasive surgery [1]. A significant majority of these deaths are preventable with surveillance of high-risk patients and early evidence-based interventions [1, 2].

Surveillance and improvement in surgical and anesthesia care is dependent on having access to continuous, reproducible, and real-time data. However, in LMICs the primary method of data capture for anesthesia and surgery is within paper health records. These records are characterized by having multiple data elements including medication administration, physiological parameters, and procedural-specific elements recorded manually by the provider at a regular frequency (e.g., every 5 min). The data density of the anesthesia paper health records, defined as the data generated per unit of time, is amongst the highest for any healthcare setting [3].

The most efficient method to record high-volume anesthesia data is with automatic data capture monitors and electronic medical record systems (EMRs). Unfortunately, due to their cost and complexity, electronic records remain an unlikely solution in LMICs for the foreseeable future [4]. This creates major gaps in digital data access for anesthesia providers in LMICs, and their ability to utilize data to rapidly anticipate and intervene to reduce anesthesia and surgical complications and mortality.

In this paper we describe our methodology to further improve the accuracy of the digitization of anesthesia paper health records from the University Teaching Hospital of Kigali (CHUK) in real time using computer vision. Our work builds from our previous digitizing efforts and further consolidates the process using a single software program. Our overarching goal for this project is to provide rapidly accessible, digital data to anesthesia healthcare providers in LMICs, which can faciliate evidence-based actionable interventions to reduce morbidity and mortality.

The remainder of this paper begins with an introduction to the paper anesthesia record from CHUK, leading into a discussion on our methodology for correcting common distortions in smartphone images of the paper anesthesia record, followed by our methods for extracting the blood pressure, physiological, and checkbox data elements. Finally, we assess the improvements in our methods from previous research in the results section, and discuss the impact, challenges, and future directions of our results and work.

### The intraoperative anesthesia paper health record

**Fig. 1:**
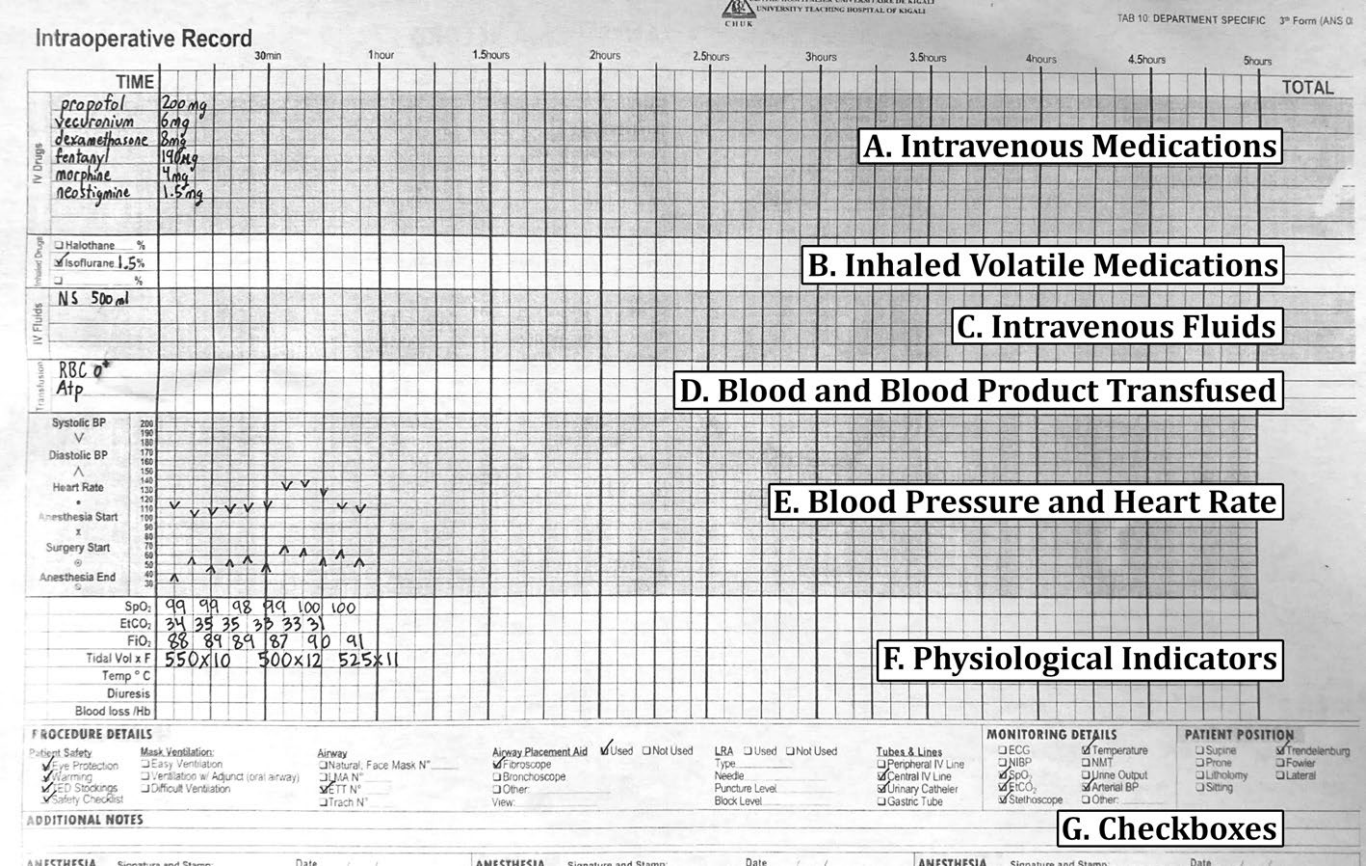
An example of an intraoperative paper anesthesia record from the University Teaching Hospital in Kigali, Rwanda

We utilized 500 smartphone photographs of paper anesthesia records collected from 2019 to 2023. The photographs of the anesthesia paper records varied greatly in quality, with some being clear, well lit, and legible, whereas others were blurry, poorly lit, and illegible. The anesthesia record has seven distinct sections: handwritten medications (Fig 1, Section A), inhaled volatile anesthetics (Fig 1, Section B), intravenous fluids (Fig 1, Section C), blood and blood product transfused (Fig 1, Section D), blood pressure and heart rate (Fig 1, Section E), physiological data elements (Fig 1, Section F), and checkboxes for marking key procedural events (Fig 1, Section G).

#### Intravenous medications

Multiple intravenous medications are administered over the course of surgery, with both the dose and timing of administration recorded in the anesthesia paper health record. Commonly administered medications include drugs required for induction of anesthesia, prevention of infection (e.g., antibiotics), to induce or reverse muscle paralysis, and to ensure blood pressure and heart stability. The medications are written in the temporal order in which they are administered.

#### Inhaled volatile medications

The inhaled volatile anesthetic medications are halogentated hydrocarbon gases that are administered to maintain general anesthesia. To document the type of the volatile inhaled anesthetic administered, the anesthesia paper health record has three checkboxes, two are for the most commonly used inhaled anesthetics: isoflurane and halothane, and the third box is a fill-in if another gas such as sevoflurane or desflurane is used. The dose of the volatile inhaled anesthetic medication is recorded as a percentage value.

#### Intravenous fluids

Intravenous fluids are administered during anesthesia to maintain fluid homeostasis and hemodynamic stability. The type of intravenous fluids, in addition to the incremental and total volume given during anesthesia is recorded as free text.

#### Blood and blood product transfused

Blood and component blood products are administered when significant bleeding and hemorrhagic complications occur. The Blood and Blood Product Transfused section is a free text section where providers list both the specific blood component product (e.g., packed red blood cells or fresh frozen plasma) and volume administered.

#### Blood pressure and heart rate

The blood pressure and heart rate section utilize handwritten arrows and dots to encode blood pressure in millimeters of mercury (mmHg) and heart rate in beats per minute (bpm). The x axis on the grid indicates five minute epochs, during which a provider takes a systolic blood pressure (downward arrow), diastolic blood pressure (upward arrow), and heart rate measurement (dot). The y-axis encodes both bpm and mmHg in increments of 10.

#### Physiological indicators

The physiological indicators section uses handwritten digits to encode different types of physiological information including oxygen saturation, inspired oxygen concentration, exhaled carbon dioxide, mechanical ventilator data, body temperature, amount of urine produced, and blood loss encountered. The x-axis on the grid represents five minute epochs.

#### Checkboxes

The checkboxes section uses handwritten check marks to indicate boolean values associated with a patient’s position on the operating table, intubation status, type of monitoring devices and details, and safety best-practices utilized during the surgery.

### Related work

In 2015, Ohuabunwa et al. [5] detailed the need for electronic medical record systems in LMICs. According to their analysis, the rise of “communicable diseases necessitates adequate record keeping for effective follow-up”, and for retrospective research. Among the difficulties with implementing these EMRs in LMICs are unfamiliarity with these systems and the cost of implementation and maintenance which make them prohibitively expensive. The authors assert that even hybrid paper-electronic systems where an image of the health record is scanned into a database and certain data elements are manually entered into an EMR can be very costly and require significant human and monetary resources. We postulate that a system which would only require the user to take a smartphone image of an anesthesia paper record would impose minimal burdens to the existing clinical workflow and require a very small amount of capital to adopt in comparison to EMR systems.

In 2020, Rho et al. described using computer vision software to automatically digitize portions of an anesthesia paper record from CHUK using smartphone images [6]. Their work utilized a wooden box within which the anesthesia paper record would be inserted and on top of which a smartphone could be placed to attain an image that was standardized for lighting and position. They digitized the checkboxes section with 82.2% accuracy, blood pressure data with an average mean squared error of 21.44 between the systolic and diastolic symbols, and classified handwritten images of medication text with an accuracy of 90.1%. It is unclear how comparable this metric is to future work, since the algorithm used was trained to reject “unreadable” samples, and did so on approximately 15% of the test set.

Subsequently, Adorno et al. developed an improved approach for blood pressure symbol detection utilizing U-Nets [7]. By generating a segmentation mask of the blood pressure symbols, using image morphology to separate the detections, and computing the centroid of each pixel cluster, Adorno was able to improve the object detection precision to 99.7% and recall to 98.2%. The mean average error of the association between U-Net detections and the ground truth blood pressure values was approximately 4 mmHg. Our approaches build on this conceptual basis of using deep learning to identify handwritten symbols in conjunction with a post-processing algorithm to associate values with detections. We implement two of the suggestions in the future work section of Adorno’s paper, namely to incorporate image tiling, and to improve the post-processing algorithms.

For checkbox detection, Murphy et al. utilized a deep neural network approach. They used a template matching algorithm called ORB and a convolutional neural network (CNN) to locate and classify the checkboxes rather than the proportion of pixel intensity method initially used by Rho et al. [8]. Their new algorithm was capable of locating checkboxes with an accuracy of 99.8% and classifying them as checked or unchecked with an accuracy of 96.7%. In subsequent development, we simplified this process by using the YOLOv8 single shot detector to combine the detection and classification steps.

Finally, Annapareddy et al. investigated the use of the YOLOv5 single shot detector to extract and classify handwritten intravenous medications and digitize the physiological indicators Sect. [9]. Due to the large number of classes in the medication and physiological indicator sections, their paper found that models that attempted both detection and classification were generally unable to do either due to lack of sufficient data in each class. However, models trained on a single class performed much better in detection, but could not classify.

## Methods

The extraction of data from an anesthesia paper chart begins with optimizing the lighting of the smartphone photographs, removing shadows, and using object detection to find document landmarks for use in removing perspective distortion. Then, each section of the chart is identified by a YOLOv8 model and cropped out of the chart. YOLOv8 models which are trained to detect handwritten blood pressure symbols, numbers, and checkboxes used in anesthesia paper charts produce lists of bounding boxes that a combination of convolutional neural networks, traditional computer vision, machine learning, and algorithms then use to impute meaningful values and detect errors.

### Image optimization techniques

To maximize the accuracy of digitization, the input images need to be optimized as follows: (1) shadows removed, (2) pixel intensities standardized and normalized, (3) perspective distortions such as rotation, shear, and scaling corrected, and (4) general location of document landmarks fixed. We accomplish this by first removing shadows using image morphology techniques, then normalize and standardize the pixel values of the images, and finally correct perspective distortions and approximately correct the location of document landmarks using a homography transformation.

#### Shadow removal

Smartphone photographs of the anesthesia paper chart often suffer from sudden changes in pixel intensities caused by shadows being cast onto the image which break up the lighting. Sudden changes in the value of pixels can cause difficulty for deep learning models which learn representations of objects as functions of the weighted sums of pixels. Therefore, both normalization and shadow removal are necessary to optimize our inputs and maximize detection accuracy. One algorithm for accomplishing this is outlined by Dan Mašek in a stack overflow post from 2017 (Algorithm 1) [10].

**Algorithm 1:**
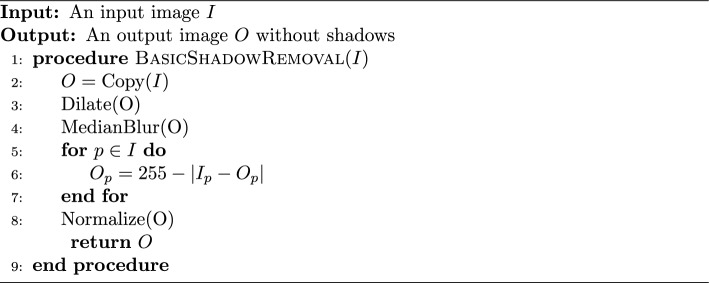
Basic Shadow Removal

**Fig. 2:**
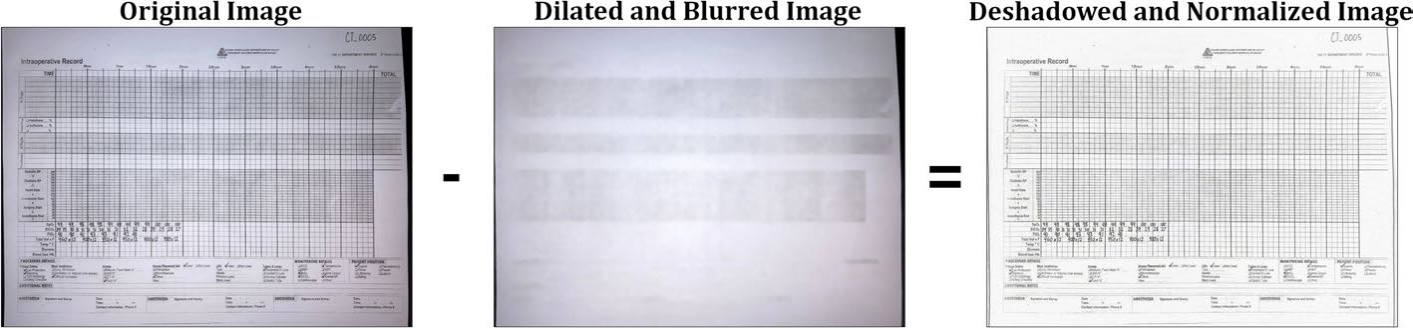
Example of an anesthesia paper chart before and after the removal of shadows and normalization. The dilated, blurred image is subtracted pixel-wise from the original image to produce the final result

The exact values for the median blur and dilation operations are subject to the image’s size and degree of shadow and can be tuned to the dataset. This algorithm only operates on grayscale images, but since no information in the anesthesia paper charts are encoded with color, we converted our charts to grayscale. We did not use any metrics to assess shadow removal, but a visual inspection of the output shows that the resulting images no longer suffer from a lighting gradient (Fig. 2).

### The planar homography

**Fig. 3:**
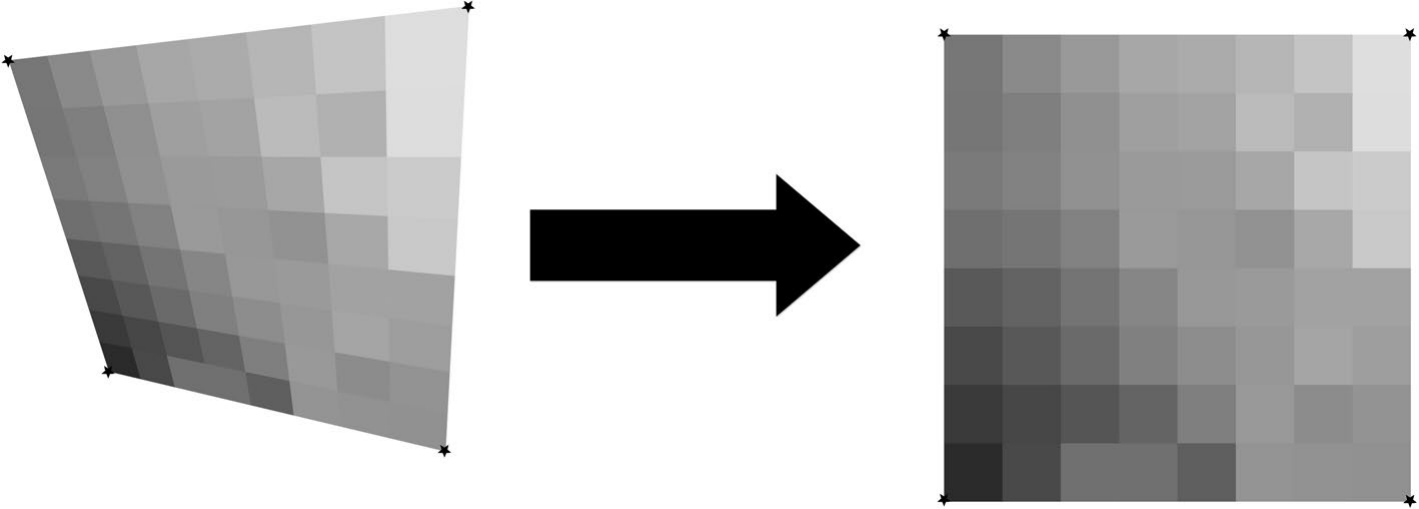
An illustration of a homography performing a general linear mapping of the points of one quadrilateral to another. Images suffering from perspective distortions can have much of their error corrected by finding four anchor points on the image, and using them as the four points on a quadrilateral to map to a perfect, scanned sheet

The planar homography is defined as the most general linear mapping of all the points contained within one quadrilateral to the points of another quadrilateral (Fig. 3). A planar homography was used to correct perspective distortions within the smartphone image.

**Fig. 4:**
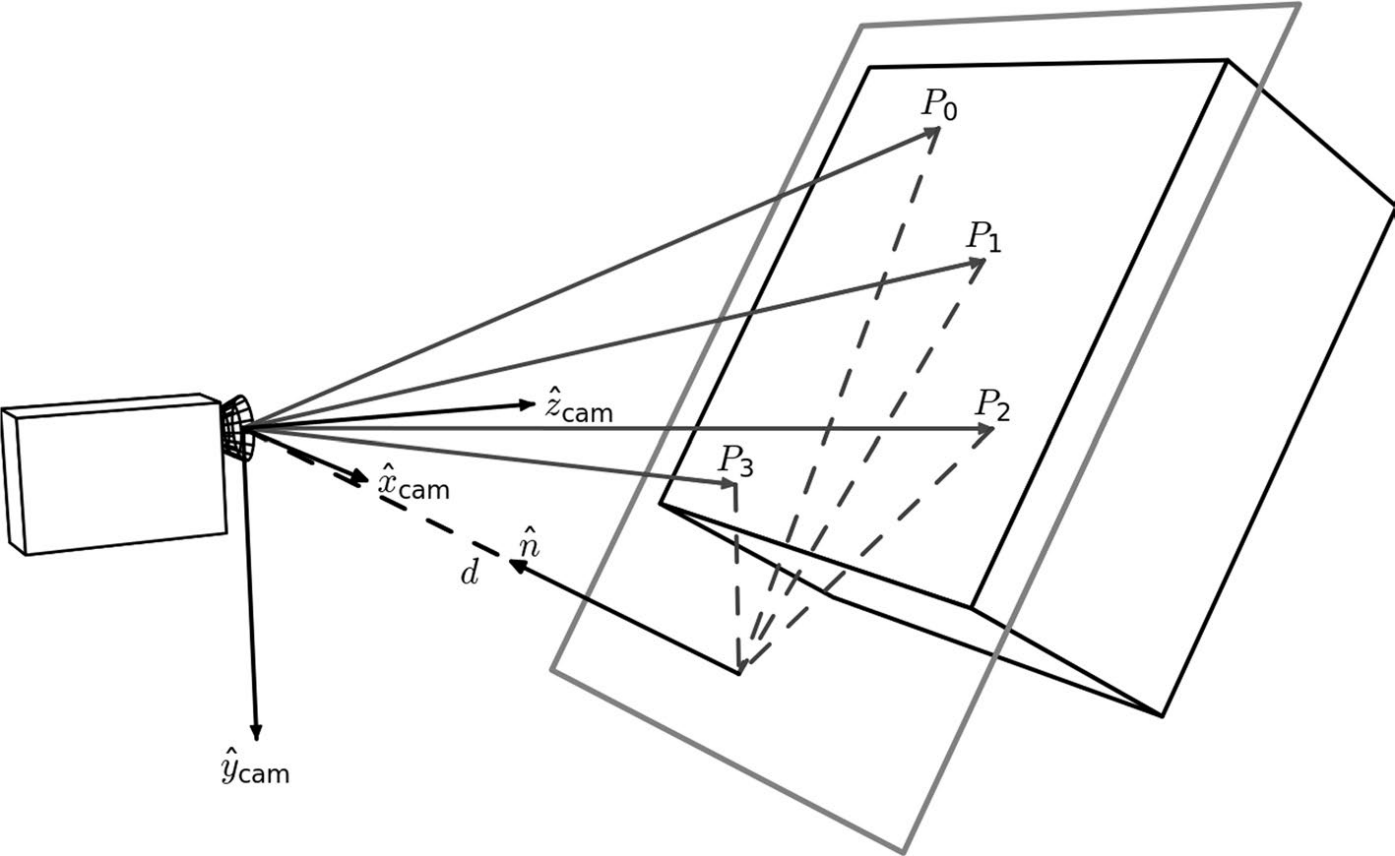
An illustration of perspective based distortion due to an off-angle camera. Even the most vigilant camera operators will have some degree of perspective distortion. [11]

Translation, rotation, scaling, affine, and shear transformations are all subsets of the homography, and the homography in turn can be decomposed into these transformations. Here, as in many other computer vision applications, the homography is used to correct linear distortions in the image caused by an off-angle camera perspective (Fig. 4).

**Fig. 5:**
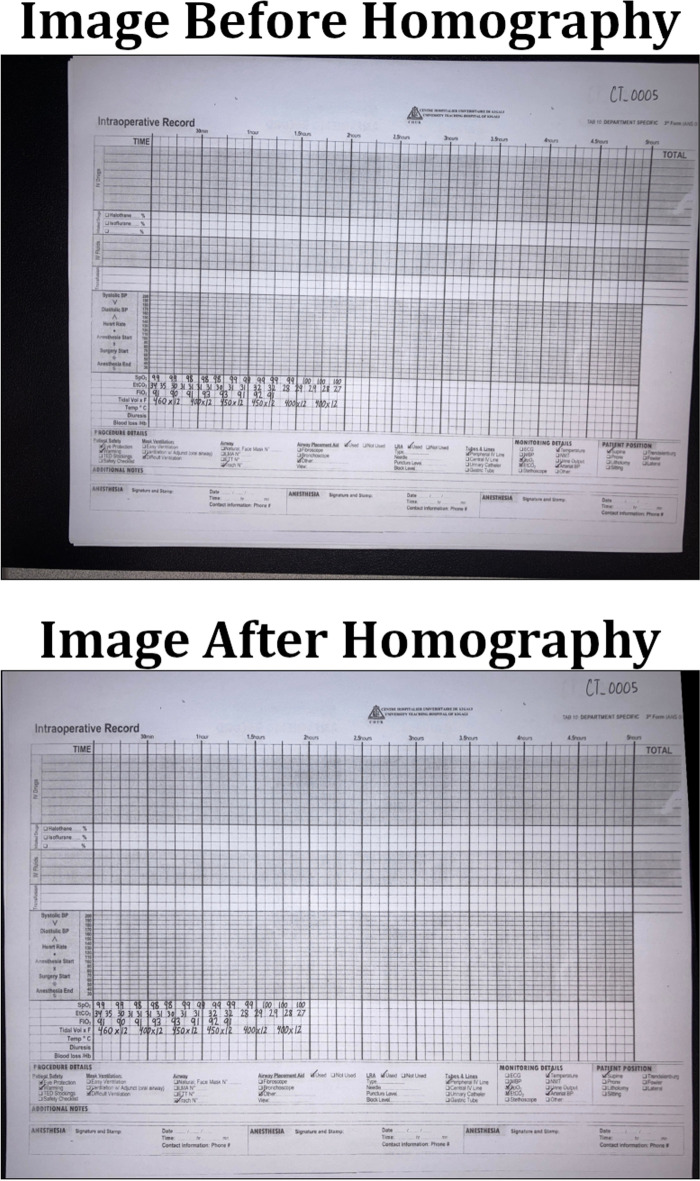
An illustration of correction using a homography on an image of the anesthesia paper chart. Perspective based distortions are corrected

In order to compute a useful homography for document correction, four document landmarks need to be identified from a target anesthesia paper chart image. Those same four landmark locations were then identified on a scanned, perfectly aligned control anesthesia paper chart image. We trained a YOLOv8 model to detect the document landmarks “Total”, “Time”, “Procedure Details”, and “Patient Position” which fall in the four corners of the anesthesia paper chart described in Fig. 1. We then used the OpenCV python package to compute the homography between the two sheets and warp the target image accordingly (Fig. 5). The benefits to this method are that the homography computation is robust to failure due to YOLOv8’s high accuracy, even under sub-optimal conditions. In cases where the planar homography failed to correct the distortion, clear errors were found on the anesthesia paper chart including: (1) landmarks being obscured by writing (2) landmarks being covered by other pieces of paper (3) landmarks not being included in the smartphone image entirely. Initially, this deep object detection approach seems excessive, as there are a number of traditional computer vision methods for automatic feature matching between two images such as ORB and SIFT. However, the variance in lighting and blurriness in our dataset posed challenges for these nondeep algorithms, which often failed silently, mistaking one landmark for another, and warping images such that they were unidentifiable.

### Section extraction

There are seven sections which encode different pieces of intraoperative information on the anesthesia paper chart (Fig. 1). Due to nonlinear distortions in the image, the homography is not a perfect pixel-to-pixel matching from the target image to the scanned control image. Therefore, an alternative method of identifying the precise location of the sections is required. We accomplished this by training a YOLOv8s model to place a bounding box around each section. Because the homography already normalizes the locations of the sections to within a few dozen pixels, we were able to train one of the smallest architectures of YOLOv8, YOLOv8s, to extract the different sections.

### Image tiling for small object detection

The anesthesia paper chart is characterized by having handwritten symbols (e.g., medication, numerical and blood pressure symbols) that are small and often tightly packed together (Fig. 1). Single shot detectors like YOLO struggle to separate and identify these handwritten symbols due to their use of a grid which assigns responsibility of a single cell to the center of a single object. One solution to this issue is to increase the image size, however since YOLO uses padding to make all images square, and the number of pixels in a square image grows quadratically with image size, this causes training memory usage and detection time to increase quadratically as well. To overcome this problem, we used an approach called image tiling where we divided the image into smaller pieces called tiles and trained on the tiles rather than the entire image. This allowed us to increase the size of these small objects relative to the frame, allowing us to get much better object detections.

**Fig. 6:**
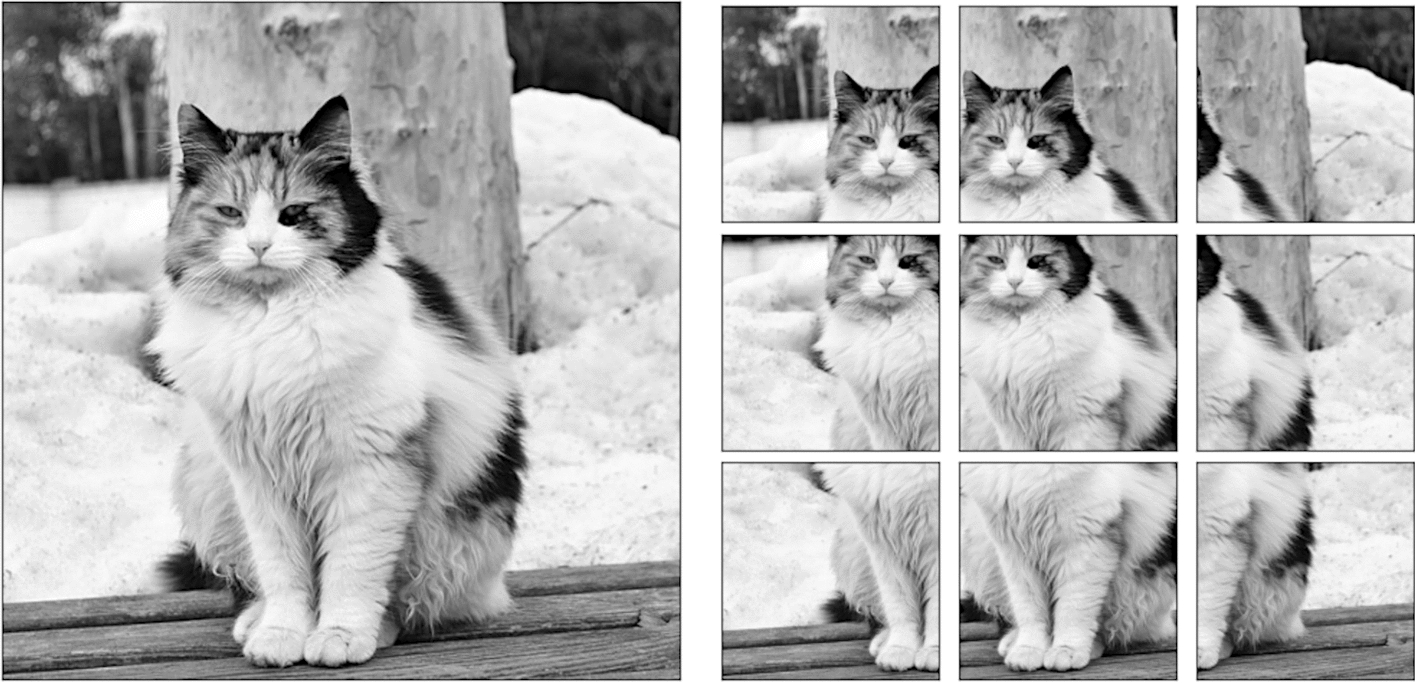
An example of our implementation of image tiling. By using a sliding window rather than a grid, the edge of one image is the center of the next one [12]

There are, however, several challenges associated with image tiling. First, objects which are larger than the tiles which we have divided the image into will not be able to fit into a single tile, and will be missed by the model. All the handwritten symbols in our dataset were small, and were uniform in size, allowing us to use image tiling without the risk of losing any detections. Second, by needing to detect on every sub-image, the detection time increases. Whereas this may be an issue in real-time detection, the difference in detection time is only measured in several hundred milliseconds, which does not affect our use case. Third, the number of unique images and total objects in a single training batch will be smaller, causing the models weights to have noisy updates and require longer training. We solved these issues by utilizing the memory savings acquired by tiling to double the training batch size from 16 to 32. In addition, due to the very large number of empty tiles, we were able to randomly add only a small proportion to the training dataset, which further increased the object to tile ratio. Finally, objects which lie on the border of two tiles will not be detected since they do not reside in either image. Our solution to this issue is to not divide the image into a strict grid, but instead to treat the tiling process as a sliding window which moves by one half of its width or height every step. With this approach, if an object is on the edge of one sub-image, it will be directly in the center of the next one (Fig. 6). This solution introduces its own challenge though since nearly every detection will be double counted when the detections are reassembled. Our solution to this problem is to compute the intersection-over-union of every bounding box with every other bounding box at detection time, group together boxes whose intersection-over-union is greater than a given threshold, and combine them into one detection. Since the objects we are detecting should be well separated and never overlap, this allows us to remove the doubled detections.

### Blood pressure symbol detection and interpretation

The blood pressure section encodes blood pressure values using arrows, and heart rate using dots or lines. Each vertical line on the grid indicates a five minute epoch of time during which a provider records a blood pressure and heart rate reading (Fig. 1). The y-axis encodes the value of blood pressure in mmHg, and each horizontal line denotes a multiple of ten (Fig. 1).

#### Symbol detection

Systolic blood pressure values are encoded by a downward arrow, and diastolic blood pressure values are encoded with an upward arrow. The downward and upward arrows are identical when reflected over the x-axis, so we were able to collapse the two classes into one. We then trained a YOLOv8 model on the single “arrow” class, and during detection we simply detect on the image and an upside-down-version of itself to obtain systolic and diastolic detections respectively. Finally, the  diastolic detections y-values are subtracted from the image's height to correct for the flip.

Thereafter two key pieces of information are required from each of the bounding boxes: (1) its value in millimeters of mercury (mmHg), and (2) its timestamp in minutes.

#### Inferring mmHg values from blood pressure symbol detections

**Fig. 7:**
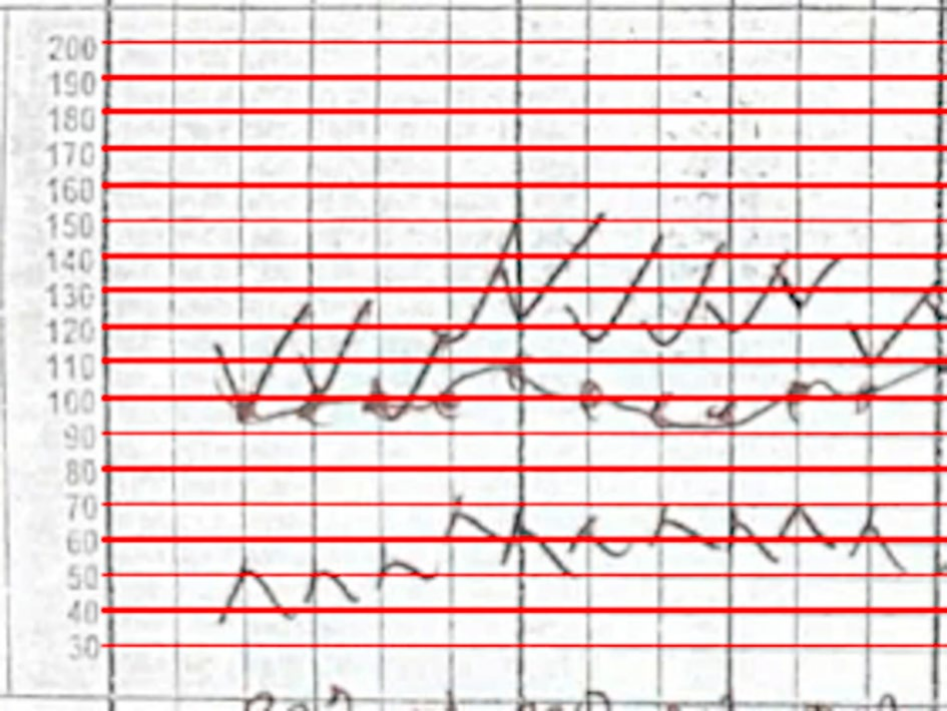
By dividing the space between the 30 and 200 bounding boxes equally, we can find the blood pressure values of each y-pixel. We ran the algorithm on this image, and set all the y-pixels that were multiples of 10 to red. We can see the efficacy of the algorithm visually as the detections cover the lines on the image almost perfectly

The value of blood pressure encoded by an arrow corresponds to the y-pixel of the tip of the arrow. By associating a blood pressure value to each y-pixel in the blood pressure section, we can obtain a value for each blood pressure bounding box. We trained a YOLOv8 model to identify the 200 and 30 legend markers, and by identifying the locations of the 200 and 30 markers, we were able to interpolate the value of blood pressure for each y-pixel between the 200 and 30 bounding boxes (Fig. 7).

#### Assigning timestamps to blood pressure symbol detections

To impute timestamps, we wrote an algorithm that applies timestamps based on the relative x distances between the systolic and diastolic detections (algorithm 2).

**Algorithm 2:**
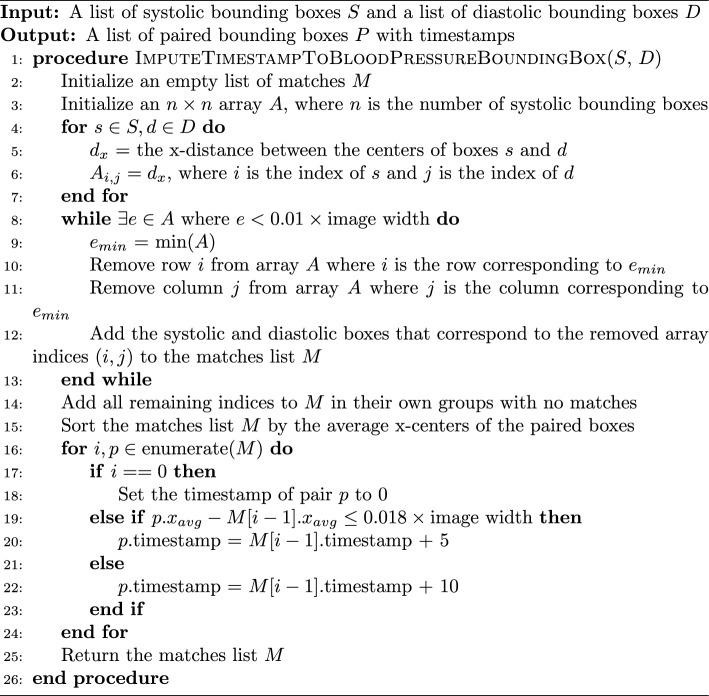
Imputing a Time Stamp to a Blood Pressure Bounding Box

Missing detections are a common problem when applying timestamps. Our algorithm deals with this in two ways. The while loop checks if two boxes are within 1% of the image’s width from one another, ensuring they are not too far away to plausibly match before actually pairing them. If a box has no pair which is within the 1% range, the algorithm considers it to not have any matches. Another problem occurs when there are no detections for a five minute epoch. This is solved by sampling the distance between true matches in the dataset. We found that 100% of the matches were within 0.016*image’s width of the next matching pair. So, adding a small amount for error, if a match is more than 0.018*image’s width from the next pair, a time gap of 10 min is applied instead of the typical 5.

#### Blood pressure model training and error testing

A YOLOv8l model, the second largest architecture of YOLOv8, was trained to detect downward arrows for 150 epochs and using a batch size of 32 images. The images used to train this model were tiled images of the blood pressure section where only the systolic arrows were annotated on unflipped images, and only the diastolic arrows were annotated on flipped images.

There are two ways that error will be assessed for the blood pressure section: detection error and inference error. Detection error will be computed using the normal object detection model metrics of accuracy, recall, precision, and F1. Inference error is the error between the value in millimeters of mercury the program assigned to a blood pressure detection on the whole image of the blood pressure section, and the ground truth value that was manually annotated. Blood pressure detections made by the program were hand matched with ground truth values during assessment in order to avoid the case where the correct blood pressure value was assigned to a different timestamp. The error metric we used for this was mean average error. The 30 chart images used for testing included 1040 systolic and diastolic marks (this number varies from the object detection testing set due to image tiling duplicating detections). The ability of the program to match blood pressure detections to a particular time stamp was not assessed.

### Physiological indicators

The physiological indicators section is the most difficult and challenging section to digitize. Handwritten digits are written on the line that corresponds to the physiological data they encode, but are free to vary along the time axis rather than being discretely boxed in, or being listed in fixed increments. In addition, the individual digits which appear in the physiological indicators section must be concatenated into strings of digits to form the number the provider intended to write. Our approach to digitize this section is described below:

#### Handwritten number detection

Our approach for the detection of numbers is a two-step process: (1) a YOLOv8 model trained on a single “digit” class which locates and bounds handwritten numbers, and (2) a RegNetY_1.6gf CNN that classifies those digits. There are two advantages to this method over using a single YOLOv8 model for both detection and classification. First, the distribution of digits in our training dataset was not uniform. For example, there are over one-thousand examples of the number ’9’ on the training charts, but only approximately 160 examples of the number ’5’ due to the typical range of oxygen saturation being between 90 and 99. This leads to the number 5 having much poorer box recall in a model that does both classification and localization. Visually, handwritten numbers are very similar to one another, so by collapsing each digit into a single “digit” class, the model can learn information about how to localize handwritten digits for numbers which are underrepresented by using numbers which are overrepresented. Second, there is an added advantage of training the classification CNN separately since the dataset can be augmented with images of digits not found on the anesthesia paper charts. We used the MNIST dataset to expand and augment our training dataset, providing sufficient examples from each class to attain a high accuracy [13].

#### Matching each box to the corresponding row

**Fig. 8:**
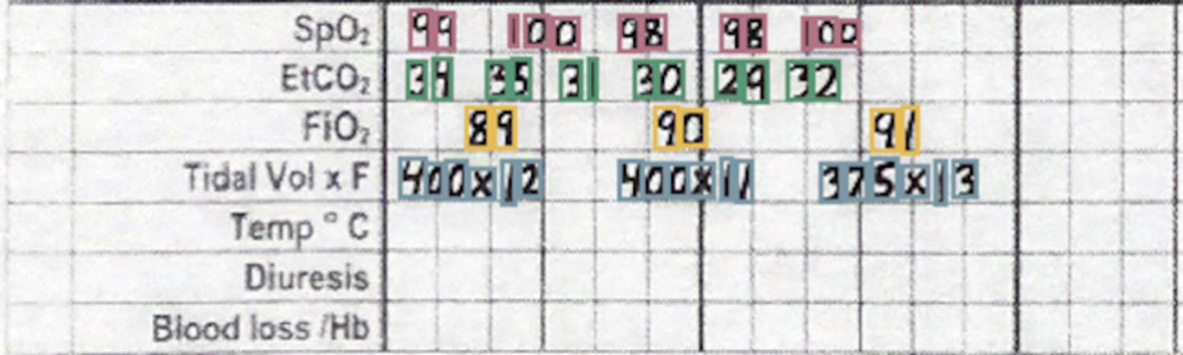
Clustered detections in the physiological indicator section using the KMeans clustering algorithm, and selecting K based on the maximum silhouette score

**Fig. 9:**
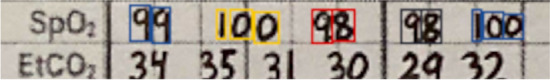
Boxes from the SpO_2_section clustered into observations using KMeans. A plausible range of values for *k* is determined by computing the number of boxes divided by the highest and lowest plausible number of digits found in a cluster (3 and 2 for the SpO_2_ section, respectively). From this range, the *k* which maximizes the silhouette score is chosen

Prior to clustering the digit bounding boxes together by proximity (Fig. 9), we had to find which row the box belongs to. For any given patient, between 0 and 7 rows were filled out depending on the type of surgery and ventilation parameter data recorded by the anesthesia provider. For the special cases where 0 or 1 rows were filled out, there were either no detected digits or the standard deviation of the y-center of the detected digits was only a few pixels. For the case where there was more than one row, we used KMeans clustering on the y-centers of the digit bounding boxes using $k \in [2, 3, 4, 5, 6, 7]$ and determined the number of rows by choosing the value of *k* which maximized the silhouette score, a metric which determines how well a particular clustering fits the data. In order to determine which row a cluster encodes, we examined the y-centroid of clusters from 30 sheets, and found that the distribution of y-centroids for a particular row never overlapped with any other row. This meant that there were distinct ranges of y-pixels that corresponded to a given row, allowing us to determine which row a cluster encodes by finding which range contained the y-centroid of a cluster (Fig. 8).

#### Clustering single digit detections into multi-digit detections

When we assigned each row an ordered list of boxes that correspond to it, we then clustered those boxes into observations that encode a single value (Fig. 9). This is done with the same KMeans-silhouette method used to find which rows each digit bounding box corresponds. In order to narrow down the search for the correct value of *k*, we used the plausible range of values for each row. For example, the first row encodes oxygen saturation, which realistically falls within the range $\text {SpO}_2 \in [75, 100]$. If we let *n* be the number of digit bounding boxes, the minimum number of clusters would be realized if the patient had a $100\%$ oxygen saturation for the entire surgery, leading to $k = \lfloor n/3\rfloor$. In contrast, the maximum number would be realized when the patient never had a $100\%$ oxygen saturation, leading to $k = \lceil n/2\rceil$. Allowing for a margin of error on either side of $10\%$ due to missed or erroneous detections, we fit a KMeans clustering model with each of $k \in [\lfloor n/3\rfloor - \lceil 0.1*n \rceil , \lceil n/2\rceil + \lceil 0.1*n\rceil ]$, and selected the value of *k* which maximized silhouette score. For the other physiological parameter rows, we reassessed the plausible number of digits for that specific variable and obtained a new range of *k* values. The clusters created by the optimal KMeans model are then considered to be digits which semantically combine to form one value.

The only section which does not conform to this paradigm is the tidal volume row. In this row, there is an “X” which separates a tidal volume in milliliters from the respiratory rate in breaths per minute. To detect semantic groupings of digits, we used the fact that tidal volume is nearly always three digits, and respiratory rate is nearly always two digits, with an “X” mark in the center, and made our search accordingly. A small CNN trained as a one vs rest model to detect the “X” mark was then trained to separate the tidal volume from the respiratory rate.

#### Assigning a value to each multi-digit detection cluster

We trained a RegNetY CNN model to classify images of handwritten numbers by combining the MNIST dataset with the digits from the charts we labeled. Initially the program runs the model on each digit in a cluster and concatenates them together to form a single value. However, due to the poor quality of handwriting, our test set classification accuracy was approximately 90% rather than the standard 99% or greater that is achievable with most modern CNNs using the MNIST dataset.

One way to minimize this error is to check if the value assigned is biologically plausible. The program first checks if the concatenated characters of a section fall in a plausible range for each row. For example, if SpO_2_$\not \in [75\%, 100\%]$ the program marks the observation as implausible. In addition, if the absolute difference between a value and the values immediately before or after it is larger than a one sided tolerance interval constructed with the differences we observed in the dataset, the program also marks it as implausible. For example, if an observation for SpO_2_ is truly 99, but the model mistakes it is 79, and the observations just before and after it is 98 and 100 respectively, the observation is marked as implausible since SpO_2_ is very unlikely to decrease and improve that rapidly. If an observation is marked as implausible, the program imputes a value by fitting a linear regression line with the previous two and next two plausible values, and predicts the current value by rounding the output of the regression model at the unknown value.

#### Physiological indicator model and error testing

A YOLOv8l model was trained to detect one class, handwritten digits, for 150 epochs with a batch size of 32.

A RegNetY_1.6gf model was trained on a mixture between observations cropped from the charts and the MNIST dataset. The model was validated and tested on observations only from the charts. The training set contained 88571 observations, while the validation and testing sets had 7143 observations each. The model was trained for 25 epochs and images were augmented using Torchvision’s autoaugment transformation under the ’imagenet’ autoaugment policy.

Error for object detection will be assessed with accuracy, precision, recall, and F1. Error for classifying numbers will be reported using only accuracy. The error for inferring a value from the classified object detections will be assessed using mean average error on each of the 5 physiological indicators on all 30 test charts. Using the output of the program and the ground truth dataset, we will compute the mean average error by index value of the lists. For example, let the program output be (99, 98, 97), the ground truth from the chart image be (98, 99, 100). Then the matched values are ((99, 98), (98, 99), (97, 100)), and the error would be computed as $((\Vert 99-98\Vert + \Vert 98-99\Vert + \Vert 97-100\Vert )/3))$. If the ground truth and predictions vary in length, the longer of the two lists will be truncated to the length of the shorter.

### Checkboxes

The checkbox section is a two class object detection and classification problem. Imputing a value can be made difficult if there are missing or erroneous detections.

#### Checkbox detection and classification

We labeled each checkbox from all the anesthesia paper charts in the dataset as checked or unchecked, and then trained a YOLOv8 model to detect and classify each checkbox in the image. Approximately one out of every twenty checkboxes that were intended to be checked did not actually contain a marking inside them. Instead, the marking would be placed on the text next to the box, slightly above the box, or adjacent to the box in some other location. We decided a priori to label these as checked because it was the intention of the provider to indicate the box as checked, and so that the model would begin to look to areas adjacent to the box for checks as well.

#### Assigning meaning to checkboxes

The checkboxes are arranged in columns (Fig. 1), so the algorithm for determining which bounding box corresponds to which checkbox starts by sorting the bounding boxes by x-center, then groups them using the columns that appear on the page, and sorts each group by y-center. For example, the left-most boxes “Eye Protection”, “Warming”, “TED Stockings”, and “Safety Checklist” on the anesthesia paper chart are all in the “Patient Safety” column, and have approximately the same x-center. The algorithm sorts all checkbox bounding boxes by x-center, selects the first four, then sorts them by y-value. Assuming there are no missing or erroneous boxes, these first four bounding boxes should match the “Patient Safety” checkboxes they encode.

#### Checkbox model training and error testing

A YOLOv8l model was trained to detect and classify checkboxes for 150 epochs using a batch size of 32. Error will be reported by overall accuracy, precision, recall, and F1 score. Sheets where the number of detections does not match the number of checkboxes will be removed from the error calculation, and the number of sheets where this occurred will be reported.

In addition to detection and classification, the program’s ability to correctly infer which checked/unchecked bounding box detection associates with which checkbox will be assessed. This error will be quantified with accuracy, precision, recall, and F1.

## Results and discussion

Our testing results were based on a 30 chart holdout set. The reason we report accuracy on these and not the testing sets used during YOLO training was due to image tiling duplicating many of the labels, which would provide an accuracy that does not reflect what would be seen on the whole section of the chart. While not reported, in all cases the test and validation sets had nearly identical metrics, suggesting the models were generalizing.

### Section extraction

On the 30 test charts, the model for extracting the sections of the anesthesia paper chart achieved an average box precision of 0.99, an average box recall of 0.99, and an mAP0.5-95 of 0.97. Due to the handwritten symbols being listed on the interior of the sections rather than the edges, a small error is, for our purposes, equivalent perfect model since it never cut off the important data elements in the sections.

### Blood pressure

Detection errors were computed using the full test set of 30 images, which in total had 1040 systolic and diastolic marks. Inference errors were computed using the first 5 images, which in total had 141 systolic and diastolic markers. This set is smaller because the systolic and diastolic markers were manually matched with their ground truth counterparts due to 8 erroneous extra markers and 2 missed markers.

#### Detection error

Table 1 demonstrates that our new method has a slightly lower accuracy rate. However, it is important to note that the previous method was tested on scanned, synthetic anesthesia paper chart images, whereas the new method was tested on smartphone images of anesthesia paper charts from real cases.

**Table 1:** Blood pressure YOLOv8 dataset

Set	Total images	Images with no annotations	Training instances
Training	18990	12799	31476
Validation	3870	2797	5244
Testing	2970	1842	5973

A breakdown of the dataset used to train the handwritten digit detection model. The dataset consisted of tiles from the larger images, so one marker on the larger image would appear in multiple tiles in varying locations. Test set error is not reported on this dataset, but instead on a 30 chart holdout set for more interpretable results

#### Inference error

The mean average error for the inference of a mmHg measurement to a blood pressure detection was only approximately 1.25mmHg, and did not vary greatly (Table 2). While not listed, the mean squared error also remains small, suggesting the error we observe did not come from a few very incorrect observations. Rather, the error we observed came from most observations being some small distance away from the true value.

**Table 2:** Physiological indicator YOLOv8 dataset

	Total images	Images with no annotations	Training instances
Training	10665	7883	17387
Validation	1350	1050	2060

A breakdown of the dataset used to train the handwritten digit detection model. No testing set was used for the YOLO model, and instead the model was tested on the 30 chart testing dataset

The MAE for imputing a value in mmHg to a blood pressure detection is much lower than previous methods. The MAE of the new method is within the variance that human beings assign to the handwritten symbols and is clinically insignificant.

### Physiological indicators

#### Detection error

By passing the output bounding boxes of the single class YOLOv8 model to the classification CNN, we can get an end to end detection error for the single characters. The overall accuracy was 85.2%, but this metric varied greatly between digits, primarily due to less representation in the training dataset for certain digits, and handwritten digits looking similar to each other (e.g., 7, 2, and 9).

#### Inference error

Obtaining an error for the imputed value of the physiological indicators is challenging. Approximately one out of every six characters that should be detected was not (false negative), and one out of every twenty proposed boxes was not actually a character, but was instead a percentage sign or other nondigit pen marking (false positive). In addition, there were relatively few examples of FiO_2_ (inspired oxygen concentration) and EtCO_2_ (end tidal carbon dioxide) in the test set, making their error highly dependent on the quality of the small number of sheets which did record them.

Therefore, we assessed error only on observations in which at least one character was detected, and a-priori decided to exclude those which were completely undetected. In addition, we left in any erroneous boxes that were clustered together with an observation.

We identified that handwriting quality had a very large positive effect on the inference accuracy, so to determine a best case error we created five synthetic sheets and filled them with an average of 35 plausible datapoints per sheet, and took images of them with smartphones in lighting similar to the real dataset. Table 3 contains the average and squared error for each section between the real anesthesia paper chart and the synthetic anesthesia paper chart test sheets. The inference error on the synthetic sheets was near zero and much more consistent than on the real anesthesia paper chart. The error on the real anesthesia paper chart was comparatively higher and more variable. When an application for smartphones is developed that will be used by physicians, we believe that the handwriting will improve to meet that of the synthetic sheets due to the Hawthorne effect [14].

**Table 3:** Physiological indicator RegNetY dataset

Character	0	1	2	3	4	5	6	7	8	9	Total
Number of obs from tiles	5902	4408	3924	2274	1562	1984	1193	1000	1537	4787	28571
Number of obs from MNIST	5923	6742	5958	6131	5842	5421	5918	6265	5851	5949	60000

A breakdown of the dataset used to train the handwritten digit detection model. MNIST data was only included in the training dataset, the validation and test datasets were entirely made from observations from charts. Because oxygen saturation is the most commonly monitored vital sign, and oxygen saturation is nearly always 98, 99, or 100, the dataset is skewed to include far more 0, 1, and 9 examples

### Checkboxes

1117 checkboxes from the 29 of the 30 test set images were used for assessing error. One test set image was excluded due to it being too blurry to manually annotate. The accuracy metrics in Table 4 demonstrate improvement in all measures, compared to previous approaches.

**Table 4:** Checkbox YOLOv8 dataset

	Unchecked	Checked
Training	23253	7795
Validation	2588	987
Testing	806	325

The number of instances of checked and unchecked boxes in the tiled dataset used to train, validate, and test the YOLO model

#### Detection error

Some checkboxes had markings which were not strictly inside the checkbox they were intending to mark, but were still classified as checked in the training dataset since the intention of the provider was to check them. Because of this, the model learned how to look in the space immediately around the checkbox to find markings, and was able to classify some checkboxes that did not have markings inside them (Tables 5, 6, 7 and 8).

**Table 5:** Blood pressure detection accuracy

Metric	New method	Previous method [7]
Recall	0.98	0.98
Precision	0.94	0.99
F1	0.96	0.99

Error metrics for two blood pressure detection algorithms: the new methods described in this paper and those previously reported

**Table 6:** Blood pressure inference error

	Systolic blood pressure	Diastolic blood pressure
New method MAE	1.00	1.36
Previous method MAE[7]	4.01	3.96

Blood Pressure Inference Error for systolic and diastolic blood pressure symbols. MAE: Mean Average Error

**Table 7:** Real test sheet versus synthetic test sheet error rates

	Real		Synthetic		
	*µ*	*σ*	*µ*	*σ*	
MAE	2.81	2.93	0.0	0.0	SpO_2_
MSE	42.38	58.3	0.0	0.0	SpO_2_
MAE	2.46	1.01	0.43	0.68	EtCO_2_
MSE	25.81	15.92	6.04	11.69	EtCO_2_
MAE	3.63	1.83	0.06	0.07	FiO_2_
MSE	39.91	37.48	0.09	0.12	FiO_2_
MAE	68.91	81.53	2.43	3.88	Tidal Volume
MSE	22793.37	37832.91	65.63	117.69	Tidal volume
MAE	4.98	9.13	0.35	0.7	Respiratory rate
MSE	240.21	584.85	2.45	4.9	Respiratory rate

Error rates for each row of the physiological indicators between the handwriting observed from the real anesthesia paper chart, and the synthetic anesthesia paper heath record. Sp02: Oxygen saturation, EtC02: end tidal carbon dioxide, Fi02: Inspired oxygen concentration

**Table 8:** Checkbox detection accuracy

Metric	New method	Previous method [8]
Accuracy	0.99	0.97
Precision	1.0	0.91
Recall	0.99	0.94
F1	0.99	0.92

Accuracy metrics for our methods (new method) compared to previous group’s methods

#### Inference error

To increase the accuracy of the data being extracted from the sheets, our exact implementation of the checkbox detection algorithm was written to throw an error if it did not detect the exact number of checkboxes on the sheet and no more. Our program did so on 4 of the 29 sheets in the test dataset (13.7%). Among the remaining 25 sheets, the program inferred the exact box that was being checked almost perfectly. The conditional error metrics are reported in Table 9.

**Table 9:** Checkbox inference accuracy

Metric	Value
Accuracy	0.99
Precision	0.99
Recall	0.98
F1	0.98

Among sheets that exactly the correct number of detections (25 of the 29 sheets in the test set), the program was able to correctly infer which box belonged to which detection

### Impact of image preprocessing

To assess the impact of both homography and deshadowing, errors were recomputed without them. We found the homography to raise accuracy across all metrics, while deshadowing had no effect on accuracy (Tables 10, 11, 12, 13, 14, 15).

**Table 10:** Blood pressure detection accuracy

Measure	Deshadowing and homography	Deshadowing only	Homography only	No preprocessing
Accuracy	0.925	0.796	0.931	0.809
Precision	0.941	0.879	0.945	0.889
Recall	0.983	0.894	0.984	0.900
F1	0.961	0.886	0.964	0.894

The effect of preprocessing on blood pressure mark detection. Removing the deshadowing component had little to no effect, but removing the homography caused a drastic drop in all metrics

**Table 11:** Blood pressure inference error

Measure	Deshadowing and homography	Deshadowing only	Homography only	No preprocessing
Systolic MAE	1.000	2.153	1.038	2.227
Diastolic MAE	1.356	2.520	1.340	2.589

The effect of preprocessing on blood pressure inference. Removing the deshadowing component has very little effect on error, but the removal of the homography correction had a negative effect on error

**Table 12:** Physiological indicator detection accuracy

Measure	Deshadowing and homography	Deshadowing only	Homography only	No preprocessing
Accuracy	0.853	0.856	0.883	0.883

**Table 13:** Physiological indicator inference error

Error	Deshadowing and homography	Deshadowing only	Homography only	No preprocessing
SpO_2_ MAE	2.81	2.64	3.29	2.43
EtCO_2_ MAE	2.46	5.25	11.41	6.02
FiO_2_ MAE	3.63	1.64	3.05	3.47
Tidal Volume MAE	68.91	69.19	76.82	139.59
Respiratory Rate MAE	4.98	3.44	2.66	4.91

The effect of preprocessing on inferring values from the objects detected in the physiological indicator section. The effects varied for each physiological indicator, so preprocessing likely did not affect the physiological indicator section much

**Table 14:** Checkbox detection ablation metrics

Measure	Deshadowing and homography	Deshadowing only	Homography only	No preprocessing
Accuracy	0.998	0.963	0.997	0.963
Precision	1.0	0.970	0.999	0.972
Recall	0.993	0.993	0.992	0.994
F1	0.997	0.981	0.996	0.984

The effect of removing deshadowing and the homography correction from the test set images. The effect of deshadowing is almost negligible, while the effect of the homography correction is pronounced

**Table 15:** Checkbox inference ablation metrics

Measure	Deshadowing and homography	Deshadowing only	Homography only	No preprocessing
Accuracy	0.990	0.994	0.989	0.994
Sheets skipped	4 (13.7%)	5 (17.2%)	4 (13.7%)	5 (17.2%)

Almost no accuracy decrease was observed on the inference of meaning to checkboxes when removing preprocessing, although removing the homography did cause one additional sheet in the test dataset to not have the exact number of detections needed to impute meaning to the checkboxes. This is listed in the second row as the number of sheets skipped with the percentage of the test set skipped in parenthesis

#### Blood pressure

#### Physiological indicators

The effect of preprocessing on the physiological indicator section was unclear. By removing deshadowing, the amount of numbers correctly detected raised by 3%, and removing both the homography correction and deshadowing had varying effects on the inference of a value for the detections (Tables 12, 13).

#### Checkboxes

The checkboxes showed very little performance loss when removing the deshadowing component, but did have a notable but small drop in the metrics when removing the homography correction (Tables 14, 15). Removing the homography caused an additional sheet from the test dataset to not have the correct number of detections for imputing meaning to the checkbox detections.

## Conclusion

In this manuscript we discussed the integration of previous research into one piece of software and the improvement of algorithms for extracting handwritten data from smartphone photographs of anesthesia paper health records. While electronic medical records are not a feasible solution for LMICs in the near future, we have demonstrated that it is possible to extract high quality data elements from anesthesia paper charts, utilizing locally available, low-cost resources such as a smartphone. Through the use of deep neural networks and the careful filtering and correction of their output by classical machine learning models and algorithms, we were able to improve the digitization of blood pressure and checkboxes to near perfect accuracy, under realistic photography and lighting conditions. In addition, we demonstrated that, through careful and legible handwriting, physiological data could likewise be digitized with high accuracy. Our work is an important step in improving access to data for health care providers in LMICs, and is a major advance in providing access to data for real time, point of care clinical decision support.

### Challenges and limitations

#### Image and chart quality

We have demonstrated the ability of the program to digitize multiple components of the anesthesia paper chart with high accuracy. However, as has been demonstrated with digitization of the physiological indicators, poor or illegible handwriting and image quality make extraction difficult, and is responsible for the majority of errors in the system. It is important to note that model development was done on previously archived anesthesia paper charts. We believe that in the future there will likely be a Hawthorne effect with improved handwriting quality when health care providers are aware that paper health records will be digitized [14]. This will improve the accuracy of the physiological data.

#### Single site usage

Anesthesia paper health charts are not standardized, with different hospitals having their own unique chart. This means that our current software will only work on a single version of the chart at a single hospital.

### Future work

#### Improvement of error detection and inference algorithms

For our initial implementation of the system, we kept the algorithms for imputing values to erroneous detections either (1) simple, using only linear models and filtering algorithms, or (2) left them out entirely, such as in the case of the checkboxes. The software we developed can now be used to test and compare local or nonlinear regression algorithms for imputing values, and new filtering methods for detecting erroneous values.

#### Digitization of remaining chart elements

There are several reasons why the remaining anesthesia paper chart elements remain undigitized. In our current dataset, Inhaled Volatile Medications (Fig. 1. Section B), Intravenous Fluids (Fig. 1. Section C) and Blood and Blood Product Transfused (Fig. 1. Section D) were infrequently recorded. In addition, the transfusions and intravenous fluids sections are completely free text, the heart rate encoding is not consistent with some anesthesia paper records using a dot, whereas others use a straight line, and the intravenous drugs section is particularly hard to read even for human clinicians. The inhaled anesthetics, however, could be digitized since they are simple checkboxes and digits, which are both currently readable. Other techniques for digitizing the data could also be available in the future, especially with a potentially larger training dataset. If a smartphone app implemented our code into a full system, the providers could list the drugs they used, eliminating the most difficult section while imposing only a minor amount of extra work for anesthesia providers.

#### Prospective creation of a new intraoperative sheet

Anesthesia paper health charts are not standardized, with different hospitals having their own unique chart. Immense time and effort is required to digitize one unique anesthesia paper health chart. To ensure future success for this project, our next goal is to design a standardized, machine readable, anesthesia paper chart using a collaborative effort between anesthesia providers from LMIC and computer vision engineers using a Delphi approach. By creating a chart prospectively, chart sections that are currently outside our ability to digitize accurately such as the intravenous fluids, transfusions, and intravenous drugs could be redesigned with machine readability in mind. For example, the intravenous drugs could have a three digit alphanumeric code written alongside the name of the medication, allowing the machine to accurately read drugs and circumventing the need to read handwritten words altogether. A smartphone app that sends images of charts to a server for processing could also store a medication-to-code dictionary so providers can easily look up the code of medications. Findings and knowledge gained from this work will guide future efforts to digitize paper charts from nonsurgical locations such as the emergency room, obstetrical delivery areas and critical care units.

## Data Availability

The data for this paper are not available due to the protected health information contained in the images.

## Abbreviations

BPM: Beats per minute
CHUK: University Teaching Hospital of Kigali
CNN: Convolutional neural network
EMRs: Electronic medical records
EtCO_2_: End tidal carbon dioxide
FiO_2_: Fraction of inspired oxygen
LMICs: Low-middle income countries
MAE: Mean average error
mAP: Mean average precision
mmHg: Millimeters of mercury
MSE: Mean squared error
ORB: Oriented FAST and rotated BRIEF
SpO_2_: Oxygen saturation
RegNetY 1.6gf: RNN regulated residual network Y 1.6 gigaflops
SIFT: Scale-invariant feature transform
YOLOv5: You only look once version 5
YOLOv8: You only look once version 8
YOLOv8s: You only look once version 8 small architecture

## Code availability

The code for this project can be found in the references [15].
